# Su(var)3-9 mediates age-dependent increase in H3K9 methylation on TDP-43 promoter triggering neurodegeneration

**DOI:** 10.1038/s41420-023-01643-3

**Published:** 2023-09-27

**Authors:** Marta Marzullo, Giulia Romano, Claudia Pellacani, Federico Riccardi, Laura Ciapponi, Fabian Feiguin

**Affiliations:** 1 Istituto di Biologia e Patologia Molecolari del CNR, Sapienza Università di Roma, 00185 Roma, Italy; 2 Dipartimento di Biologia e Biotecnologie “C. Darwin”, Sapienza Università di Roma, 00185 Roma, Italy; 3 International Centre for Genetic Engineering and Biotechnology, Padriciano 99, 34149 Trieste, Italy; 4 Department of Life and Environmental Sciences, University of Cagliari, 09042 Monserrato, Cagliari, Italy

**Keywords:** Chromatin remodelling, Amyotrophic lateral sclerosis

## Abstract

Aging progressively modifies the physiological balance of the organism increasing susceptibility to both genetic and sporadic neurodegenerative diseases. These changes include epigenetic chromatin remodeling events that may modify the transcription levels of disease-causing genes affecting neuronal survival. However, how these events interconnect is not well understood. Here, we found that Su(var)3-9 causes increased methylation of histone H3K9 in the promoter region of TDP-43, the most frequently altered factor in amyotrophic lateral sclerosis (ALS), affecting the mRNA and protein expression levels of this gene through epigenetic modifications that appear to be conserved in aged *Drosophila* brains, mouse, and human cells. Remarkably, augmented Su(var)3-9 activity causes a decrease in TDP-43 expression followed by early defects in locomotor activities. In contrast, decreasing Su(var)3-9 action promotes higher levels of TDP-43 expression, improving motility parameters in old flies. The data uncover a novel role of this enzyme in regulating TDP-43 expression and locomotor senescence and indicate conserved epigenetic mechanisms that may play a role in the pathogenesis of ALS.

## Introduction

Aging is associated with a series of molecular changes, that lead to functional tissue deterioration and predispose to an increased likelihood of disease and death. This process, interestingly, does not seem to happen randomly but follows a programmed sequence of events that appear to be conserved among evolutionarily divergent species [1–3]. In the nervous system, neuronal aging or senescence can be functionally quantified through two main phenotypes, the deterioration of cognitive functions and the reduction of locomotory capacities. These alterations, on the other hand, coincide with the insidious symptoms that signal the onset and progression of the most common neurodegenerative diseases such as Alzheimer’s disease (AD), Parkinson’s disease (PD), or amyotrophic lateral sclerosis (ALS) [4, 5] endorsing the idea that aging and pathological neurodegeneration may be regulated by a common set of genes [6, 7].

Molecularly, a common feature of aging is the epigenetic changes in chromatin organization that occur after the post-translational modifications of histones [8, 9]. These modifications are conserved, affect the expression parameters of numerous genes, and may provoke alterations in the expression levels of proteins that constitute risk factors for neurodegenerative diseases. In support of this view, we and others have described that the conserved TDP-43, a heterogeneous nuclear ribonucleoprotein (hnRNPs) largely associated with the pathogenesis of ALS [10–12] and permanently required in the motor system (comprising motoneurons, associated glia, and skeletal muscles) to maintain locomotor activity, becomes downregulated during aging in *Drosophila* and mammalian brains [13–21]. Even though these fluctuations in protein levels appear to be consistent and conserved in highly different species, the physiological relevance of reduced TDP-43 expression during aging, the molecules involved in its downregulation, and/or their contribution to neuronal senescence is not known. In this study, we investigated the mechanisms by which TDP-43 becomes downregulated during aging and the functional implications of these modifications in the onset and progression of motoneurons degeneration.

## Results

### Recovery of TDP-43 function during aging prevents locomotor decline

**Fig. 1:**
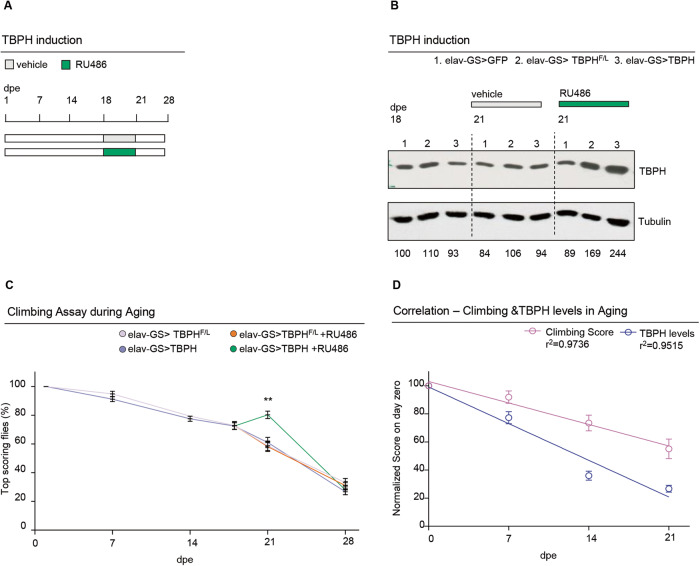
TBPH prevents locomotory senescence in *Drosophila*. **A** Schematic representation of the *elav*-Gene Switch induction protocol with RU486 (in green). The drug was added to fly food at day 18 until day 21, then the flies were transferred to standard food. **B** Western blot showing the TBPH levels in protein extracts from fly heads of the reported genotypes 1, 2 and 3 at day 18, and at day 21 in drug (RU486) or vehicle-only treated. Membranes were probed with anti-TBPH and anti-tubulin antibodies. Lane 1 = UAS-GFPmCD8/+;*elav-*GS/+; lane 2 = +/+;*elav-*GS/UAS-TBPH^F/L^; lane 3 = UAS-TBPH/+;*elav*GS/+;. Numbers below represent band quantification normalized on internal loading (tubulin). Average of two experiments. **C** Climbing assay in adult flies of the reported genotypes (+/+;*elav-*GS/UAS-TBPH^F/L^; and UAS-TBPH/+;*elav-*GS/+), without (pink and blue, respectively) or with RU486 (orange and green, respectively) induction at different days post eclosion (7, 14, 18 and 21 dpe). Each point represents the percentage of flies able to reach the top of a 50 ml tube in 10 s after being tapped to the bottom. *n* **≥** 100 animals for each genotype, in at least three technical replicates. ns, not significant; **p < 0.01 calculated by one-way ANOVA. Error bars represent SEM. **D** Graphical representation of the correlation between climbing score (pink line) and TBPH protein levels (blue line) in *w*^1118^ flies at 1,7,14 and 21 dpe. As shown in the graph both climbing score and TBPH protein levels have a significant inverse correlation (*p* < 0.05) with aging (r^2^ = 0.9736 and r^2^ = 0.9515, respectively).

Progressive degeneration in locomotor activity, also known as locomotor senescence, is one of the main phenotypes used to quantify the impact of age on the functional organization of the nervous system and negative geotaxis (the ability of flies to vertically climb a test cylinder) a well-accepted assay for measuring neuromuscular capacity in vivo [22–24]. Using this methodology, we have described that the progressive decrease in *Drosophila* locomotor activity during aging correlates with a physiological decrease in the expression of the TBPH protein, homologous to the human TDP-43 [20]. Consistently, we and others showed that also in mice TDP-43 undergoes an aging-dependent decrease [20, 21], highlighting the evolutionary relevance of this phenomenon. However, the relationships between these events have not been clarified yet. To determine whether the drop in TBPH/TDP-43 expression during aging plays any role in locomotor senescence, we used the GeneSwitch (GS) system to generate flies carrying the neuronal driver *elav*-GS-GAL4 and the transgene UAS-TBPH (*w*^1118^; UAS-TBPH/+; *elav*-GS-GAL4/+) to modulate the expression of TBPH in a temporally controlled manner by adding the RU-486 (mifepristone) activator in the fly food [19, 25, 26]. As controls, we utilized the TBPH^F/L^ allele unable to bind the RNA (*w*^1118^; UAS-TBPH^F/L^/+; *elav*-GS-GAL4/+) and the unrelated protein GFP (*w*^1118^; UAS-EGFP/+; *elav*-GS-GAL4/+) [27]. Thus, we detected that GS-flies in which the promoter was not activated, showed a significant decrease in locomotor activity around 7 days post eclosion (dpe). This diminution in fly motility increases progressively during aging (50% at 14 dpe to 30% of their initial capacity at 21 dpe) and correlates with a decrease in TBPH/TDP-43 mRNA and protein levels (Supplementary Fig. S1). Thus, to determine whether TBPH reintroduction in aged animals may prevent locomotor senescence, we induced the expression of the UAS-TBPH transgene (*w*^1118^; UAS-TBPH/+; *elav*-GS-GAL4/+) in 18 days old flies during 72 h, by adding the RU486 activator to the fly food (Fig. 1A, B). Notably, we found that induction of TBPH expression improved climbing abilities and slowed the locomotor decline in aged flies compared to UAS-TBPH^F/L^ (Fig. 1C), establishing a direct correlation between the age-related decrease in TBPH expression and locomotor deterioration (Fig. 1D).

### H3K9 methylation at the *TARDBP*/*TBPH* promoter increases with aging and is conserved in both flies and mammals

**Fig. 2:**
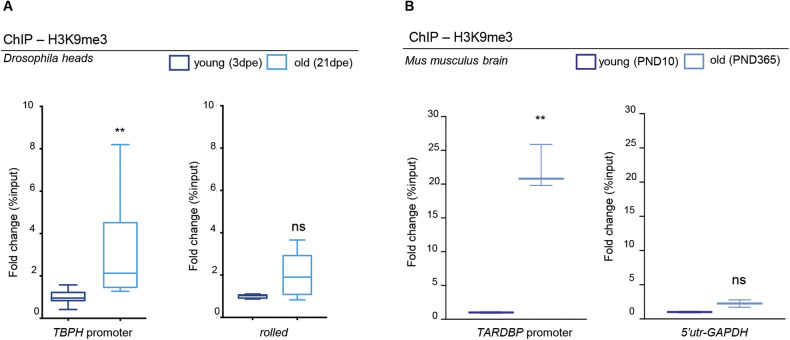
Levels of H3K9me3 at *TBPH/TARDBP* promoter increase with age. **A** qRT-PCR analysis on the *TBPH* promoter or on a control heterochromatic region (*rolled*), immunoprecipitated either with an anti-H3K9me3 antibody or with a control IgG antiserum in chromatin extracts from 3- or 20-days post eclosion (dpe) fly heads. The DNA enrichment is shown as a percentage of input DNA and normalized on the *GADPH* gene used as control. Note the significant increase (∼2 fold) of *TBPH* promoter in 21 dpe flies compared with 3 dpe. No significant changes were observed in the control gene (*rolled*). Error bars represent SEM of three independent experiments (*n* = 3; pull of 300 heads), 3 biological replicates and 3 technical replicates); **p = 0.0019, ns, not significant; Mann-Whitney t-test. **B** qRT-PCR analysis on the *TARDBP* promoter or on the *GADPH-5’UTR* gene used as control, immunoprecipitated either with an anti-H3K9me3 antibody or with a control IgG antiserum in the brain of C57 mice at post-natal day 10 (PND 10) or PND 365. The DNA enrichment is shown as a percentage of input DNA and normalized on the total H3. Note the significant increase (∼20 fold) of *m-TARDBP* promoter in PND 365 mice compared with PND 10. No significant changes were observed in the control gene (*m-GADPH*). Error bars represent SEM of three independent experiments (*n* = 6 mice per group, 3 biological replicates); **p < 0.01, ns, not significant; Mann-Whitney t-test.

Gene expression is a tightly regulated process influenced by the epigenetic modifications of the histones, that controls the accessibility to the DNA (in particular those located in promoter regions) to a large number of proteins that can directly promote the regulation of transcription [28, 29]. Mechanistically, the methylation of the histone H3K9 (H3K9me) by specific methyltransferase enzymes, constitutes the initial event that triggers the formation of repressive heterochromatin domains in the DNA [30, 31]. Thus, to determine if the downregulation of *TBPH* during *Drosophila* aging is related to changes in the methylation patterns of H3K9, we performed chromatin immunoprecipitation (ChIP) studies and assessed the binding profile of H3K9me3 on the *TBPH* promoter. Remarkably, we found a significant enrichment in H3K9me3 amounts sited on the *TBPH* promoter in chromatin samples extracted from old flies compared to young controls (Fig. 2A), revealing an increase in the levels of repressive heterochromatin modifications on the *TBPH* promoter in vivo during aging [21, 31, 32]. In support of this observation, we noted that these epigenetic changes do not appear to be due to a generalized and/or nonspecific increase in H3K9 methylation caused by age, as its overall biochemical levels decrease in old brains (Supplementary Fig. S2), suggesting that the modifications described on the *TBPH* promoter are rather specific and may promote transcriptional repression of this gene. Importantly, we observed that similar modifications in H3K9 methylation levels of the TDP-43 promoter, take place also in the mammalian brain. Thus, H3K9me3 chromatin immunoprecipitation assays in C57 mice brains at post-natal day 10 (PND 10) and PND 365, showed a very significant increase (~20 fold) in the methylation levels of the *TARDBP* promoter in old mice compared to young samples or to unrelated controls (Fig. 2B), revealing that these modifications follow well-conserved designs.

### *Su(var)3-9* mediated H3K9 methylation of the *TBPH* promoter regulates gene expression levels and locomotor aging in flies

**Fig. 3:**
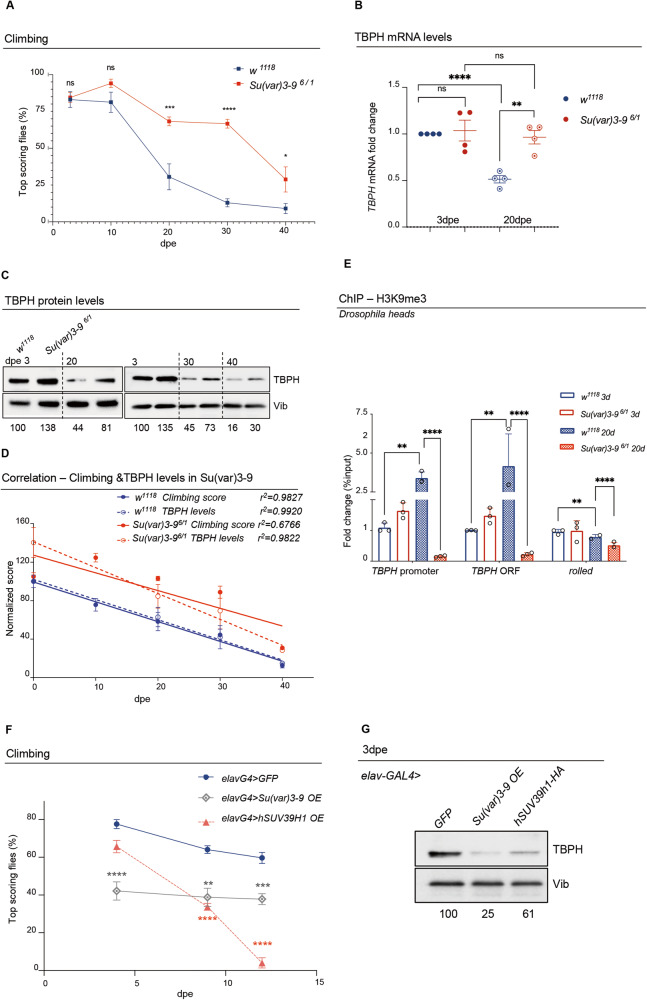
Loss of Su(var)3-9 rescues TBPH ageing-dependent decrease and associated reduced climbing abilities. **A** Climbing assay performed in *Su(var)3-9* mutant flies (*Su(var)3-9*^*6*^*/Su(var)3-9*^*1*^*;* red curve) or in control flies (*w*^*1118*^*;* blue curve), at different days post eclosion (3, 10, 20, 30 or 40 dpe). Each square represents the percentage of flies able to reach the top of a 50 ml tube in 10 s after being tapped to the bottom. *n* **≥** 30 animals for each genotype, in at least five technical replicates. ns, not significative; *p > 0.05; ***p < 0.001; ****p < 0.0001 with one-way ANOVA. Error bars represent SEM. **B** qRT-PCR showing *TBPH* mRNA levels in *Su(var)3-9* mutants [*Su(var)3-9*^*6*^*/Su(var)3-9*^*1*^*;* red] compared to controls (*w*^*1118*^; blue) in RNAs from young (3dpe; full circles) or old (20 dpe; empty-dotted circles) flies heads extracts. Error bars represent SEM of three independent experiments (*n* = 3; pull of 50 heads), 3 biological replicates and 3 technical replicates). **p < 0.01; ****p < 0.0001 with one-way ANOVA. **C** Western Blot showing the TBPH protein levels in *Su(var)3-9* mutants [*Su(var)3-9*^*6*^*/Su(var)3-9*^*1*^] compared to controls (*w*^*1118*^) in fly head extracts at different days post eclosion (3, 20, 30 or 40 dpe). Numbers below represent band quantification (the average of four experiments) normalized on internal loading (Vibrator, Vib). **D** Graphical representation of the correlation between climbing score (full dots) and TBPH protein levels (empty dots) in *w*^1118^ flies (in blue) and *Su(var)3-9*^*6/1*^ (in red) at 3, 10, 20 and 30 dpe. In the graph has been reported the correlation value (r^2^) for the climbing score and the TBPH protein levels in both genotypes (*w*^1118^ and *Su(var)3-9*^*6/1*^). As shown in the graph, the climbing score of wild type flies has a significant inverse correlation with aging (*p* < 0.05; r^2^ = 0.9827, blue line full dots); while in *Su(var)3-9*^*6/1*^ mutants the climbing score does not have a significative inverse correlation with aging (*p* = 0.0921; r^2^ = 0.6659; red line full dots). Differently, the TBPH protein levels have a significant inverse correlation with aging in both wild type (blue dashed line empty dots) and *Su(var)3-9*^*6/1*^ mutants (red dashed line empty dots) (p < 0.05; r^2^ = 0.9920 and r^2^ = 0.9822, respectively). However, the normalized scores of the *Su(var)3-9*^*6/1*^ mutants are always higher of those of the wild type. **E** qRT-PCR analysis on both the *TBPH* promoter and its coding sequence compared to a control heterochromatic region (*rolled*), immunoprecipitated either with an anti-H3K9me3 antibody or with a control IgG antisera in chromatin extracts from young (3 dpe) or old (20 dpe) *Su(var)3-9* mutants [*Su(var)3-9*^*6*^*/Su(var)3-9*^*1*^] or controls (*w*^*1118*^). The DNA enrichment is shown as a percentage of input DNA and normalized on the *GAPDH* gene used as control. Error bars represent SEM of three independent experiments (*n* = 3; pull of 300 heads, 3 biological replicates and 3 technical replicates). **p < 0.01; ****p < 0.0001 with one-way ANOVA. **F** Climbing assay performed in adult flies overexpressing UAS-*Su(var)3-9 (*gray curve*)*, or *UAS-hSuv39h1-HA* (orange curve) under the control of the *elav-GAL4* driver or in control flies expressing a *UAS-GFP* construct (blue curve), at different days post eclosion (3, 7, or 12 dpe), at 29 °C. Each dot represents the percentage of flies that reach the top of a 50 ml tube in 10 s after being tapped to the bottom. *n* **≥** 30 animals for each genotype, at least 5 technical replicates. **p < 0.01 ***p < 0.001; ****p < 0.0001 calculated by one-way ANOVA. **G** Western Blot showing the TBPH protein levels in heads extracts of flies overexpressing the *UAS*-*Su(var)3-9* or the *UAS-hSuv39h1-HA* or *UAS-GFP* under the control of the *elav-GAL4* driver at 3 days post eclosion. Numbers below represent band quantification normalized on internal loading (Vibrator, Vib).

In order to explore the physiological significance of increased H3K9 methylation in the *TBPH* promoter region, we decided to modulate the activity of Su(var)3-9, the well-described and conserved histone methyltransferases capable of methylating H3K9 in vivo [31, 33]. Strikingly, we found that null alleles of *Su(var)3-9*, in trans-heterozygous combinations (*Su(var)3-9*^*6*^*/ Su(var3-9*^*1*^), sired viable and fertile flies that present a significant increase in their locomotor capacities in adulthood compared to age-matched controls in climbing assays (Fig. 3A; Supplementary video V1). Accordingly, the locomotor performance of either 20, 30, or 40 days old *Su(var)3-9* mutant flies significantly exceeded the climbing abilities of wildtype flies of the same age. Along these lines, we quantified that the loss of locomotor capacity in *Su(var)3-9* mutant flies between 3 and 30 days after hatching (from 84% of flies reaching the top to 66%, respectively) was much less pronounced than in wildtype controls (from 83% to 12%, respectively), underlining the unexpected role of this enzyme in regulating locomotor performances and neurological senescence (Fig. 3A). Furthermore, biochemical analyses performed on fly head extracts obtained from the flies described above (3 and 20 days-old trans-heterozygous combinations *Su(var)3-9*^*6*^*/Su(var)3-9*^*1*^ or *w*^*1118*^ wildtype controls), revealed that both *TBPH* mRNA and protein levels are higher in *Su(var)3-9* mutants compared to the wildtype controls (Fig. 3B–D). Moreover, ChIP analyses, showed that *Su(var)3-9* old mutant flies presented reduced levels of H3K9 methylation in the promoter and coding regions of *TBPH* compared to controls (Fig. 3E), indicating that these molecular differences in methylation and expression levels may underlie the phenotypic changes in motility. In support of this hypothesis, we found that overexpression of *UAS-Su(var)3-9* or its human counterpart *UAS-SUV39H1*, under the control of the neuronal driver *elav*-GAL4 (Supplementary Fig. S3A), was sufficient to deeply affect the locomotor capacities of these flies, inducing early locomotor decline and provoking a strong reduction in the levels of TBPH protein expression in *Drosophila* brains (Fig. 3F, G), revealing that Su(var)3-9 plays a major role in the epigenetic control of TBPH expression. Additionally, we found that incubation of wildtype *Drosophila* brains with chaetocin (unfortunately the compound, in the present formulation, does not pass the gastric barrier to be tested in vivo) causes an increase in *TBPH* expression and a reduction in H3K9 methylation, mimicking the effect caused by the loss of *Su(var)3-9* (Supplementary Fig. S3B). Remarkably, we observed that the role of Su(var)3-9 in the regulations of *TBPH* promoter was rather specific since the loss of two additional enzymes able to methylate H3K9, like eggless and G9a in *Drosophila* [31], was unable to modify the expression levels of TBPH in fly heads or affect locomotor behaviors in vivo (Supplementary Fig. S3C–E). Curiously, we observed that the expression of the *Drosophila* homolog of Fus (dFUS-*cabeza*), a gene epistatically related to TBPH and ALS-related factor [12, 34], does not change over time (Supplementary Fig. S3F), suggesting that the age-dependent locomotor decline is specifically related to *TBPH* reduced transcription.

### The conserved SUV39H1 enzyme regulates TDP-43 expression levels in human cells

**Fig. 4:**
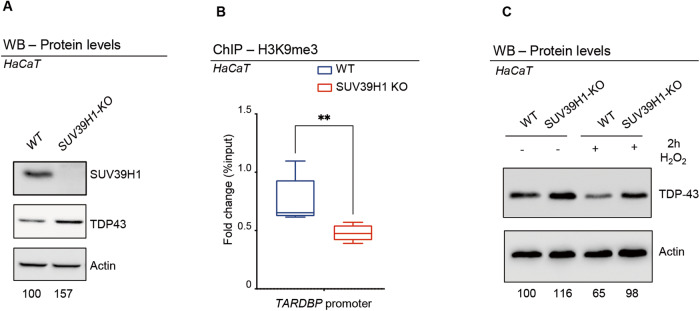
SUV39H1 depletion in human cells correlates with reduced levels of H3K9me3 at T*ARDBP* promoter and with a corresponding increase in TDP-43 protein. **A** Western Blot showing the SUV39H1 and TDP-43 protein levels in extracts from WT or SUV39H1 KO cells. Numbers below represent band quantification normalized on internal loading control (actin; average of 6 experiments). **B** qRT-PCR analysis on the *hTARDBP* promoter immunoprecipitated with an anti-H3K9me3 in chromatin extracts from WT or SUV39H1 KO cells. Enrichment is shown as a percentage of input DNA and normalized on the *GADPH* gene used as control. Error bars represent SEM of three independent experiments (*n* = 3, 3 biological replicates) **p < 0.01 calculated by Mann-Whitney t-test. **C** Western blots showing the expression levels of TDP-43 in wild type (WT) or *SUV39H1 KO* HaCaT Keratinocytes after (+) or not (−) treatment with H_2_O_2_ (200 mM) for 2 h (2 h). H_2_O_2_ treatment reduces TDP-43 levels in WT but not in SUV39H1-KO cells. Numbers below represent band quantification normalized on internal loading control (actin; average of 3 biological repetitions).

To determine if the conserved SUV39H1 histone methyltransferase is able to regulate the methylation of the TDP-43 promoter and modulate protein expression also in human cells, we took advantage of a HaCaT cell line carrying a CRISPR-Cas9 mutation in the *SUV39H1* gene (*SUV39H1 KO*; [35]. Interestingly, we observed that in these cells the absence of SUV39H1 causes an increase in the levels of TDP-43 protein expression (Fig. 4A). In the same direction, H3K9me3 ChIP analyses revealed a significant reduction in H3K9me3 amounts sited on the *TARDBP* promoter in chromatin samples extracted from SUV39H1 KO cells compared to wildtype cells (Fig. 4B), suggesting that epigenetic modifications mediated by SUV39H1 might be responsible for the transcriptional repression of *TARDBP* and, above all, underlining the remarkable conservation found in the regulation of this locus. To challenge whether aging-induced modifications would also play a role in the regulation of human TDP-43, we treated wildtype or SUV39H1 KO cells with H_2_O_2_ a classic and well-accepted treatment for inducing cellular senescence [36–38]; (Supplementary Fig. S4). As a result, we found that H_2_O_2_ induced a significant reduction in TDP-43 protein expression which is prevented by the deletion of the *SUV39H1* gene (Fig. 4C), indicating that similar age-dependent regulatory mechanisms might be present in human cells.

## Discussion

One of the most fundamental features of aging is the progressive deterioration in locomotor skills. Despite some studies, in both mice and flies revealing that TDP-43/TBPH levels decrease during aging [20, 21, 39], the functional significance of this reduction in protein expression has not been established, nor have the mechanisms involved in the regulation of these modifications been identified. In this manuscript, we found that induction of the TDP-43 fly counterpart, TBPH, expression in old fly neurons, but not of the TBPH^F/L^ mutated form (unable to bind RNA), is sufficient to rescue locomotor senescence, demonstrating a direct correlation between these events and revealing a novel role for TDP-43/TBPH in the regulation of age-dependent locomotor degeneration. In that direction, alteration in the function of TDP-43 is considered one of the main causes of ALS and it has been shown that pathological variations in the intracellular levels of this protein (both gain or loss of function) were able to cause neurodegeneration, indicating that tight control of TDP-43 expression is crucial to prevent neurological phenotypes [40–42]. These observations, therefore, highlight the importance that the knowledge of novel genes or molecules capable to modulate TDP-43 activity could have for understanding the pathogenesis of ALS [43–46]. According to that, we found an age-dependent increase in H3K9 methylation at the TBPH/TDP-43 promoter region mediated by *Su(var)3-9* in *Drosophila* and confirmed that these modifications are conserved in mice brains and human cells. Moreover, we established that these regulatory mechanisms were sufficient to modulate the expression levels of TDP-43 in both flies and human cells and to affect locomotor behaviors. Interestingly, a similar outcome was detected using chaetocin, a chemical compound capable of inhibiting Su(var)3-9-mediated H3K9 methylation [47]. These data reinforce the idea that Su(var)3-9 plays a fundamental role in the epigenetic regulation of *TBPH* expression and identifies a compound capable of regulating the expression levels of this gene in situ, contributing to the development of potential pharmacological interventions against ALS or locomotor weakening in the future.

**Fig. 5:**
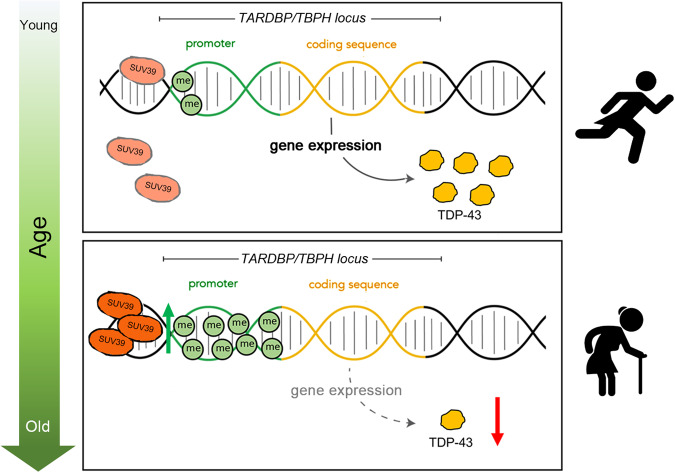
Schematic representation of the mechanism of action of Suv39 at *TARDBP/TBPH* promoter region during aging. SUV39 activity at the *TARDBP/TBPH* promoter region is increased in elderly individuals. This effect results in increased methylation of H3K9 on *TARDBP/TBPH* locus, leading to reduced levels of TDP-43 expression and diminished locomotor capabilities.

To further investigate how aging affects the methylation status of the TBPH/TDP-43 promoter, we have also examined the expression levels of three well-conserved H3K9 demethylase genes: Kdm3 (CG31123), Kdm4A (CG15835), and Kdm4B (CG33182) by conducting qPCR analysis on brain extracts of wild-type flies at 3- and 20-days post eclosion (dpe). Our findings demonstrated an age-related increase in the expression levels of Kdm4A and Kdm4B, while the expression of Kdm3 remained unchanged (Supplementary Fig. S3H). The data indicate that the demethylases we examined may not directly contribute to the observed increase in methylation at the TDP-43 promoter during aging and suggest the involvement of alternative mechanisms beyond the overall state of chromatin methylation or demethylation in the epigenetic regulation of specific loci, such as TBPH/TDP-43 (Supplementary Fig. S2). Intriguingly, we discovered that the accumulation of aging-related factors, such as H202 in cultured human cells can induce early senescence and leads to a reduction in TDP-43 protein expression, which appears to be mediated by the human-homolog gene SUV39H1 (Fig. 4C). These results suggest that the metabolic changes preceding and driving aging could potentially modify or enhance the function of SUV39H1 in specific regions or loci of the chromosome [48, 49]. Supporting this notion, ChIP array analyses conducted in *Drosophila* brains demonstrated the increased accumulation of the *Su(var)3-9* protein at the promoter region of *TBPH* during aging [48, 50]. Furthermore, we detected a significant upregulation of *Su(var)3-9* expression in aged flies (Supplementary Fig. S3G), illustrating how the gradual accumulation of epigenetic modifications in this locus can occur over time (Fig. 5). Similar positive outcomes have been observed in previous studies where targeting SUV39H1, either through pharmacological or genetic inhibition, resulted in improvements in memory and learning abilities in aged mice, accompanied by the restoration of neuronal gene BDNF expression [51]. Additionally, SUV39H1 inhibition has been found to protect mice from myocardial infarction by preventing SIRT1 transcriptional repression [52]. These findings underscore the potential therapeutic implications of modulating Su(var)3-9/SUV39H1 activity, suggesting that it could be a viable strategy for mitigating age-related locomotor and cognitive decline and addressing other age-associated disorders. Further research is necessary to explore the underlying mechanisms and assess the translational potential of SUV39H1 as a therapeutic target modulator.

In conclusion, we have identified an unprecedented mechanism whereby Suv39 regulates the epigenetic status of the *TARDBP/TBPH* promoter and drives the progression of locomotor aging through the regulation of TDP-43/TBPH expression levels. This role of *Su(var)3-9* seems to be evolutionarily conserved from *Drosophila* to vertebrates and may contribute to understanding the interrelationships between human aging and neurodegenerative diseases.

## Methods

### Drosophila strains and rearing conditions

Drosophila stocks were maintained on standard fly food (25 g/L corn flour, 5 g/L lyophilized agar, 50 g/L sugar, 50 g/L fresh yeast, 2.5 mL/L Tegosept [10% in ethanol], and 2.5 mL/L propionic acid) at 25 °C in a 12 h light/dark cycle. All experiments were performed in the same standard conditions, otherwise differently specified. The following fly strains were purchased from the Bloomington Drosophila Stock Center (BDSC, Indiana University, Bloomington, IN, USA): *w*^*1118*^ (BDSC #3605); *elav-GS* (BDSC #43642); *UAS-TBPH* (BDSC #93601); *UAS-TBPH.F-L* (BDSC #93781); *UAS-mCD8-GFP* (BDSC #30002); *Su(var)3-9*^*1*^*/TM3* (BDSC #6209); *UAS-Su(var)3-9.lacI* (BDSC #93147); *UAS-hSUV39H1.HA* (BDSC #84799); *elav-GAL4* (BDSC #77894); *egg*^*1473*^*/SM1* (BDSC #30565). The *Su(var)3-9*^*6*^*/*TM6B allele was a kind gift of Gunter Reuter [49], the *G9a*^*RG5*^ allele was a kind gift of Marion Delattre [53].

### Climbing assays

The locomotion activity was measured by quantification of geotactic response. Equal ratio of male and females of the desired ages will be transferred, without anesthesia, to a 50 ml conical tube, tapped to the bottom of the tube, and their subsequent climbing activity quantified as the percentage of flies reaching the top of the tube in 10 s [51]. The number of climbing events was scored for 5 consecutive times. Flies were assessed in batches of 15, at least three biological replicates were performed for each condition [18]. In all climbing experiments each group of flies was analyzed for climbing ability at all different age points (3-10-30-40 dpe) and then sacrificed (at 40 dpe). Extracts for western blots at 3-20-30 were obtained from extra groups of flies. These flies were always analyzed also for climbing activity before being used for extracts.

### RU486-Induction protocol

The Gene Switch system was activated by adding the RU486 (Sigma-Aldrich #M8046) activator to the fly food. A stock solution of 50 mM RU486 in 95% ethanol was diluted to the final concentration of 0.5 mM in 2% sucrose and the solution was been added on the surface of standard cornmeal medium to feed adults.

### Chaetocin treatment

Adult fly heads or larval brains were separated from the bodies and incubated with 100 nM chaetocin (Sigma-Aldrich #C9492) or 100% Ethanol in Schneider’s Medium supplemented with 10% FBS for 2 h at room temperature. Heads were then washed in PBS1x and collected for subsequent analysis.

### Chromatin immunoprecipitation

#### Fly heads

Heads of frozen flies were separated by vortexing for 15 s and isolated using 630 μm and 400 μm sieves. 400–600 fly heads were homogenized in homogenization buffer [350 mM sucrose, 15 mM HEPES pH 7.6, 10 mM KCl, 5 mM MgCl2, 0.5 mM EGTA, 0.1 mM EDTA, 0.1% Tween, with 1 mM DTT and Protease Inhibitor Cocktail (PIC, Roche) added immediately prior to use] at 4 °C. The homogenate was fixed using 1% formaldehyde for 10 min at RT and then quenched with glycine. The tissue debris was removed by filtration with 60 μm nylon net (Millipore). Nuclei were collected and washed with RIPA buffer at 4 °C (150 mM NaCl, 25 mM HEPES pH 7.6, 1 mM EDTA, 1% Triton-X, 0.1% SDS, 0.1% DOC, with protease inhibitors added prior to use). The extract was sonicated 6 times with 2 min cycles (Branson Sonifier 250, output = 50%). Sonicated samples were centrifuged for 10 min at 12,000 × *g*. Two hundred and fifty micrograms of chromatin DNA were subjected to a 1 h preclearing with 50 *μ*l of a 50% protein G-Sepharose (GE healthcare) bead slurry containing 1% BSA. Before the Immunoprecipitation, 5% of the total extract was collected as INPUT. The precleared samples were then immunoprecipitated overnight with 5 *μ*g of anti-H3K9me3 (Abcam ab8898) or anti-rabbit IgGs (Sigma, 15006) at 4 °C. The immune complexes were incubated for 4 h at 4 °C with 50 *μ*l of fresh protein G-Sepharose beads. After immunopurification, beads were washed four times with RIPA and once with LiCl wash buffer (250 mM LiCl, 10 mM Tris-Hcl pH 8.0, 1 mM EDTA, 0.5% NP-40, protease inhibitors PIC (Roche). Beads were re-suspended in TE buffer and incubated ON at 65 °C. Proteins were digested with Proteinase K (10 mg/ml) at 55 °C for 1 h. Immunoprecipitated DNA was purified using Phenol:Chlorophorm:Isoamyl alchol extraction. Immunoprecipitated DNA and 5% input DNA were analyzed by SYBR-Green real-time qPCR. The run was performed by using the Applied Biosystems (Waltham, MA) Quant-Studio 3 Real-Time PCR System 36 instrument. Primer Sequences described previously are reported in Table S1.

#### Mouse brain

Chromatin immunoprecipitation in brain of C57 mice at post-natal day 10 (PND 10) and PND 365 was performed using EpiQuik Tissue Chromatin Immunoprecipitation kit (Epigentek #P-2003) according to manufacturer’s instructions. Briefly, 150 mg of frozen tissue were cut into small pieces (<1 mm^3^) and cross-linked with 1% formaldehyde for 10 min at room temperature and then quenched in PBS 1X-Glycine 1.25 M for 10 min at room temperature. The samples were homogenized using a Douncer homogenizer and centrifuged to pellet nuclei. After homogenization, lysis buffer was added to nuclei. Chromatin was prepared and sonicated using a water bath Bioruptor (Diagenode; 30” ON/30” OFF, High power, 3 × 10 cycles) to a size range of 200–1000 bp. To pre-cleared cell debris, sonicated chromatin was centrifuged at 12,000 x g at +4 °C for 10 min. Chromatin was diluted and ChIP performed according to manufacturer’s instructions using antibodies against H3K9me3 (ab8898, Abcam), histone H3 (ab1791, Abcam), IgG1 (G3A1, Cell Signalling) was used as negative control in the immunoprecipitation. Immunoprecipitated DNA was purified by phenol-chloroform extraction and in parallel 5 ul (5%) were taken to be used as input in the quantification analysis. qPCRs were performed using iQ SYBR Green in a CFX96 Real-Time PCR system (Bio-Rad). Primer sequences are reported in Table S1.

#### Human HaCaT cells

HaCaT cells were crosslinked with 1% formaldehyde fixing buffer (1% Formaldehyde; 5 mM Hepes pH8.0; 0.05 mM EGTA pH 8.0; 10 mM NaCl) at 37 °C for 10 min and then quenched with glycine, rinsed twice with cold phosphate-buffered saline, and then lysed and harvested in ChIP lysis buffer (50 mM Tris-HCl pH 8.1; 0.5% SDS; 10 mm EDTA;100 mM NaCl, 1 mM PMSF, Proteinase inhibitor Roche) by centrifugation for 6 min at 2000 × *g*. Cells were then resuspended in sonication buffer (50 mM Tris-HCl pH 8.1; 10 mm EDTA; 1% Triton-X; 0,1% deoxycholate sodium; 100 mM NaCl, 1 mM PMSF, Proteinase inhibitor Roche) and sonicated 6 times with 2 min cycles (Branson Sonifier 250, output = 50%). Sonicated samples were centrifuged for 10 min at 12,000 × *g* and the supernatant were diluted 5-fold in sonication buffer. Two hundred and fifty micrograms of chromatin DNA were subjected to a 1 h preclearing with 50 *μ*l of a 50% protein G-Sepharose (GE healthcare) bead slurry containing 1% BSA. Before the Immunoprecipitation, 5% of the total extract was collected as INPUT. The precleared samples were then immunoprecipitated overnight with 5 *μ*g of anti-H3K9me3 (Abcam ab8898) at 4 °C. The immune complexes were then incubated for 4 h at 4 °C with 50 *μ*l of fresh protein G-Sepharose beads. Following incubation, the beads were collected by centrifugation for 1 min at 2000 × *g* and washed consecutively for 3–5 min with 1 ml of each solution: low-salt wash buffer (0.1% SDS; 1% Triton X-100; 2 mM EDTA; 20 mM Tris pH 8.1; and 150 mM NaCl), high-salt wash buffer (0.1% SDS; 1% Triton X-100; 2 mM EDTA, 20 mM Tris pH 8.1; and 500 mM NaCl), LiCl wash buffer (250 mM LiCl; 1% NP-40, 1% deoxycholate sodium salt, 1 mM EDTA, and 10 mM Tris pH 8.1), and twice in Tris and EDTA buffers (10 mM Tris pH 8.1 and 1 mM EDTA). Immune complexes were then eluted with 120 *μ*l of buffer containing 1% SDS and 100 mm NaHCO_3_. Crosslinking was reversed by incubating the samples overnight at 65 °C. Proteins were digested with Proteinase K (10 mg/ml) at 55 °C for 1 h. Immunoprecipitated DNA was purified using Phenol:Chlorophorm:Isoamyl alcohol extraction. Immunoprecipitated DNA (1.5 μl) and 5% input DNA were analyzed by SYBR-Green real-time qPCR (as described in Antonucci et al. 2014). The run was performed by using the Applied Biosystems (Waltham, MA) Quant-Studio 3 Real-Time PCR System 36 instrument. Primer Sequences described previously are reported in Table S1.

### RNA extraction and quantitative PCR

Total mRNA was isolated from *Drosophila* adult heads by using Trizol reagent (15596026, Thermo Fisher Scientific) according to the manufacturer’s instructions. RNA was reverse-transcribed (1 mg each experimental point) by using SensiFAST cDNA Synthesis Kit (BIO-65053, Bioline) and qPCR was performed as described [52] using SensiFast Sybr Lo-Rox Mix (BIO- 94020, Bioline). The run was performed by using the Applied Biosystems (Waltham, MA) Quant Studio 3 Real- Time PCR System 36 instrument. Primer Sequences are reported in Table S1.

### Human HaCAT cells

The immortalized human epidermal keratinocyte (HaCaT) cell line was obtained from [35]. The HaCaT cells were cultured in complete media, which comprised of Dulbecco’s modified Eagle’s medium (DMEM) supplemented with 10% (v/v) heat-inactivated fetal bovine serum and 1% (v/v) penicillin-streptomycin at 37 °C in a humidified atmosphere of 5% CO_2_/95% air.

### H_2_O_2_ treatment

HaCaT cells (10^5^cells) were cultured on 35 mm cell culture dish for 24 h and treated with H_2_O_2_ at 200 μM/l for 2 h at 37 °C.

H_2_O_2_ was washed with PBS for terminating the treatment. Cells were kept on the incubation in normal medium for another 24 h. Cells were then harvested and assessed in western blot.

### Western blot

#### Fly extract

Protein extracts were derived from adult fly heads, lysed in sample buffer or Urea Buffer (150 mM NaCl, 10 mM Tris-HCl pH8, 0,5 mM EDTA, 10% glycerol, 5 mM EGTA, 50 mM NaF, 4 M urea, 5 mM DTT, Protease Inhibitor Cocktail (PIC) (Roche), fractionated by SDS-PAGE and transferred to nitrocellulose membrane. Primary antibodies were: anti-TBPH rabbit (1:1000; homemade [18]); anti-Actin goat (1:1000; Santa Cruz, sc-1616); anti-Vibrator rabbit (1:5000; also named Giotto [54]); anti-H3K9me2 mouse (1:400; Abcam ab1220), anti-H3K9me3 rabbit (1:1000; Abcam ab8898); anti-Tubulin mouse (1:5000; Sigma, T-5168); anti-HA HRP (1:1000; Santa Cruz sc7392); anti-Su(var)3-9 rat (1:50; [33]). As a secondary antibody, we used the appropriate HRP-conjugated antibody (GE Healthcare) diluted 1:5000 in PBS-Tween 0.1%. Membranes were incubated 5 min with ECL substrate (#1705062 and #1705060, Bio-Rad) and the HRP-ECL reaction was revealed using the ChemiDoc^TM^ XRS gel imaging system (Bio-Rad). Band intensity quantification was performed using the gel analyzer tool in Fiji/ImageJ software.

#### HaCAT extract

Cells were harvested and centrifuged at 5000 rpm for 5 min at 4 °C. The supernatant was removed, Buffer WCE 2X (100 mM TrisHCl pH 6.8, 4% SDS, 200 mM DTT) was added to resuspend the cell pellet, boiled for 10 min and then added an equal volume of SDS-PAGE Sample Loading Buffer [2X] (100 mM TrisHCl pH 6.8, 4% SDS, 200 mM DTT, 20% glycerol, 0.004% bromphenol blue) to the mixture. Cell extracts were pelleted at 15,000 g in an Eppendorf centrifuge for 15 min at 4 °C and the supernatants were analyzed by Western blotting according to [55], using the following antibodies, all diluted in TBS-T: anti-p-p53 (Ser 15; 1:1000, Santa Cell Signaling), anti-p53 (1:1000, Santa Cruz), anti-p-H2AX (Ser 139; 1:1000, Millipore), anti-SUV39H1 (44.1; 1:1000, Santa Cruz Biotechnology), anti-TDP-43 (1:5000, Proteintech), anti-H3k9me3 (1:1000, Abcam ab8898), anti-H3K9me2 (1:500, Abcam ab1220), anti-actin-HRP-conjugated (1:5000, Santa Cruz Biotechnology). These primary antibodies were detected using HRP conjugated anti-mouse and anti-rabbit IgGs and the ECL detection kit (all from GE Healthcare). Band intensities were quantified by densitometric analysis with Image Lab software (Bio-Rad).

All full length uncropped original western blots are available in the Supplementary Materials section.

### Statistical analysis

Statistical analysis was performed using Prism six software (MacKiev). The Shapiro-Wilk test was used to assess the normal distribution of every group of different genotypes. Statistical differences for multiple comparisons were analyzed with the Kruskal-Wallis for non-parametric values or with one-way ANOVA for parametric values. The Dunn’s or the Tukey’s test was performed, respectively, as post hoc test to determine the significance between every single group. The Mann-Whitney U-test or the t-test were used for two groups’ comparison of non-parametric or parametric values, respectively. Pearson coefficient was used to assess correlation. A *p* < 0.05 was considered significant. In climbing trials, the number “n” represents a group consisting of a minimum of 15 flies. In biochemical or molecular assays, “n” represents the number of biological replicates or the number of extracts that have been independently generated and analyzed.

### Supplementary information

Su(var)3-9 mediates age-dependent increase in H3K9 methylation on TDP-43 promoter triggering neurodegeneration

Supplementary video V1

## Data Availability

All data generated or analyzed during this study are included in this published article and its supplementary information files. Any additional request is available from the corresponding author on reasonable request.
