# Supplementary material for: Su(var)3-9 mediates age-dependent increase in H3K9 methylation on TDP-43 promoter triggering neurodegeneration
Source: Cell Death Discov. 2023 Sep 27;9:357. doi: 10.1038/s41420-023-01643-3 (PMC10533867; doi:10.1038/s41420-023-01643-3)
Supplement: Supplementary file 1 — Su(var)3-9 mediates age-dependent increase in H3K9 methylation on TDP-43 promoter triggering neurodegeneration [file 41420_2023_1643_MOESM1_ESM.pdf]

Supplementary Materials

Su(var)3-9 mediates age-dependent increase in TDP-43 promoter methylation triggering neurodegeneration

Table S1. List of primer used in this work:

Name	Sequence	Reference
TBPH_real-time FW	CGGCAAGCCGAGCACGATGAG	(1)
TBPH_real-time RV	CGCGGAGTTCGCTCCAACGAG	
TBPH_promoter FW	CACTAGCCCTGTTCGGCCAC	This study
TBPH_promoter RV	AGCATTTCTTCTCGCTTTCCGTT	
rolled_FW	TTGGATTGGCTCGTATTGCA	This study
rolled_RV	CCATCGGGTAGCAACGTATTC	
GAPDH_FW	CCTGGCCAAGGTCATCAATG	(2)
GAPDH_RV	ATGACCTTGCCCACAGCCTT	
TDP-43_promoter FW (mouse)	GAAGCCAGTGGGAGAGG	This study
TDP-43_promoter RV (mouse)	ACAACAGCCGCGCTACC	
GAPDH-5'UTR FW (mouse)	GGGTTCCTATAAATACGGACTGC	(3)
GAPDH-5'UTR RV(mouse)	CTGGCACTGCACAAGAAGA	
caz(dFus)_FW	GCAATTTGTACGCAGCGAGT	This study
caz(dFus)_RV	TGGCTCGTTGTAGTTACCCG	
human_TARDBP_promoter FW	CAACCGGTGGGAGAGGACGCCG	This study
human_TARDBP_promoter RV	CGCGACTCACCCGCTAGGCCG	
*Su(var)3-9*_FW	CCACGGTGGTCAAAGCCATA	This study
*Su(var)3-9*_RV	GGCTATGTCGCCGCAATTC	
TBPH_ORF FW	TTCCCAAGGGTAAATTCGAG	This study
TBPH_ORF RV	TGGCAGTTGAGTTCTCCAAG	
Actin FW	AAGCTGTGCTATGTTGCCCT	(4)
Actin RV	ATTCCCAAGAACGAGGGCTG	
Kdm3 FW	TAGTTGCGCTTCGCTCAGTG	This study
Kdm3 RV	AGCCGCCAATTCTTTTTGCG	
Kdm4A FW	TGAAAGGTCAGGACATGGGC	
Kdm4A RV	CGGATTTGGCTGCACGTAAG	
Kdm4B FW	GCTAGTGTATTGTGTGTTGTTCC	
Kdm4B RV	CGACCTGTTGTCGGACACTT	

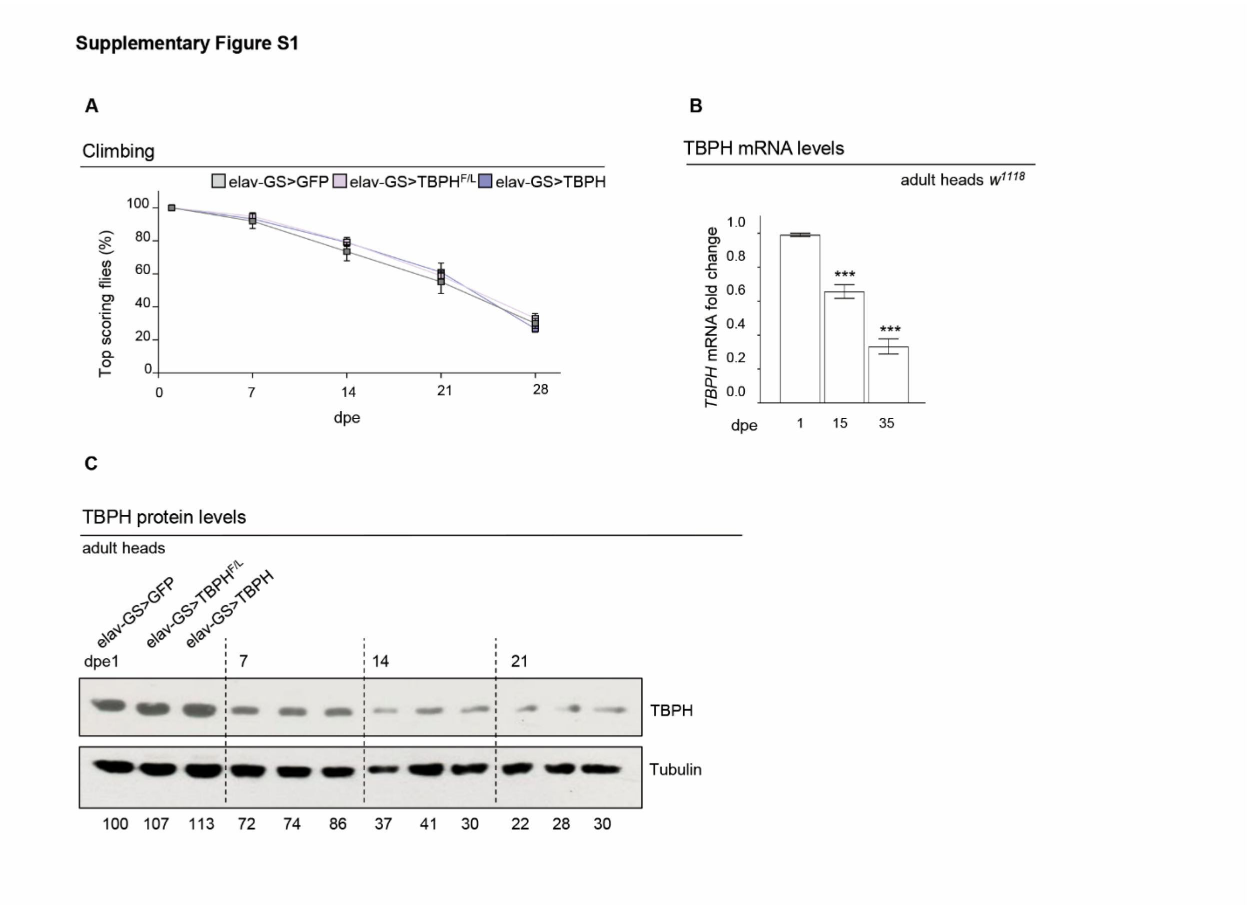

Supplementary Figure S1. Locomotor decline correlates with reduction of TBPH levels in Drosophila

(**A**) Climbing assay performed on adult flies of the reported genotypes at different days post eclosion (7, 14, 21, or 28 dpe) without induction with RU486 of the *elav*-GeneSwitch GAL4 driver. Each box represents the percentage of flies able to reach the top of a 50 ml tube in 10 seconds after being tapped to the bottom. n≥ 80 animals for each genotype, in at least three technical replicates. ns, not significant with one-way ANOVA. Error bars represent SEM. (**B**) qRT-PCR showing *TBPH* mRNA levels in RNAs from wild type (w^{1118}) fly head extracts. Error bars represent SEM of 3 biological replicates. *** p<0.001, by one-way ANOVA. (**C**) Western Blot showing the TBPH levels in wild type (w^{1118}) protein extracts from fly heads of *elav*-GeneSwitch flies driving UAS-GFP (1), UAS-$TBPH^{F/L}$ (2) or UAS-TBPH (3) without RU486 induction at different days post eclosion (7, 14 and 21 dpe). Numbers below represent band quantification normalized on internal loading (tubulin).

Supplementary Figure S2

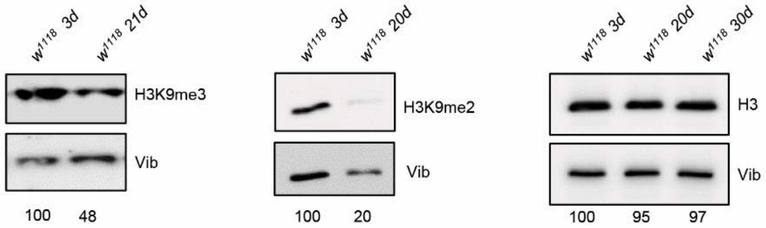

Supplementary Figure S2. Global H3K9 methylation decreases during aging.
Western Blot on protein extracts from adult fly heads at 3 or 20dpe detected with anti-H3K9me3, anti-H3K9me2 and anti-H3 showing that the global levels of H3K9 methylation undergo an aging-dependent reduction, while the total histone H3 does not change. Numbers below represent band quantification normalized on internal loading (Vibrator, Vib). Average of two experiments.

Supplementary Figure S3

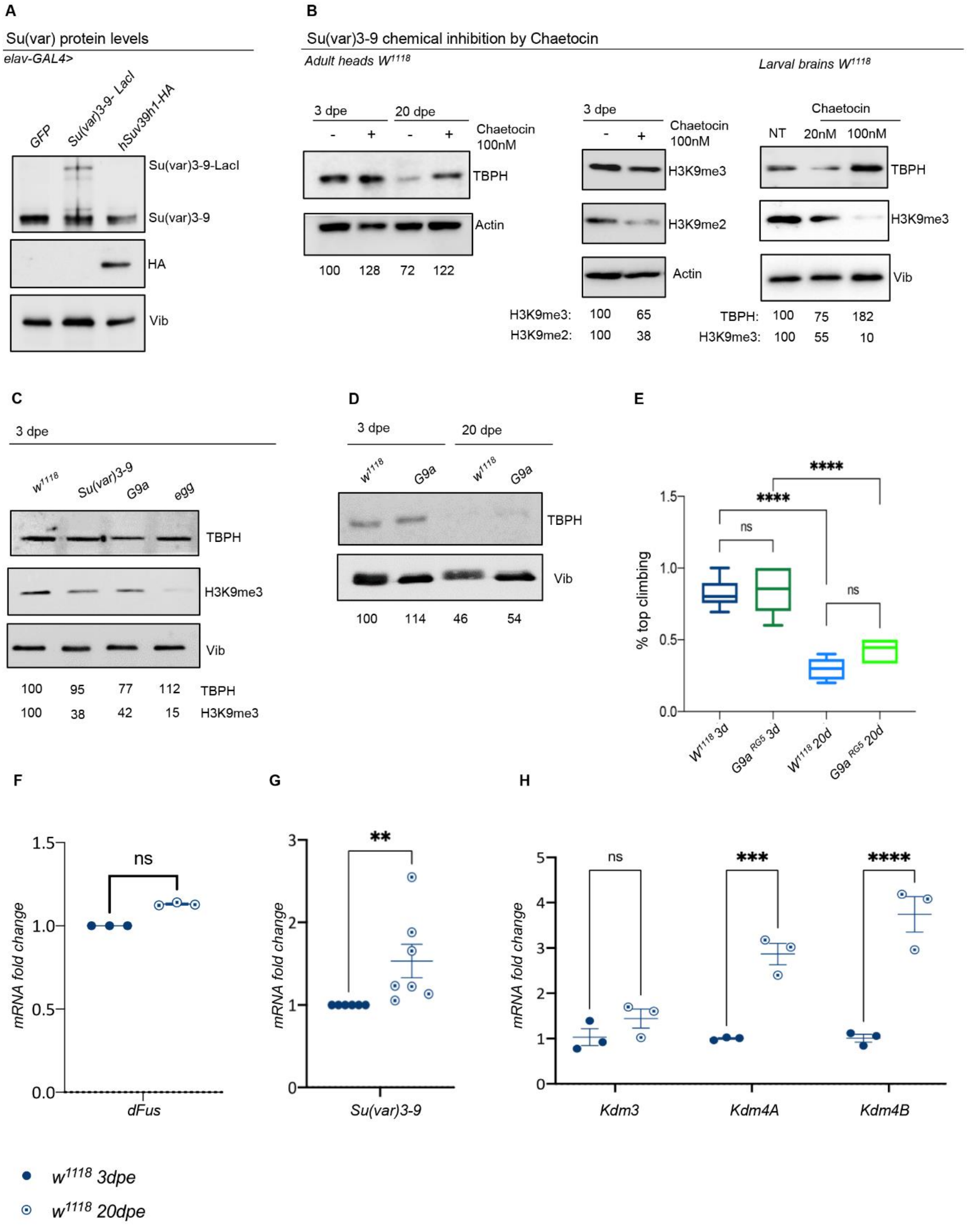

Supplementary Figure S3. Su(var)3-9 specifically controls TBPH expression levels.

(**A**) Western Blot showing the SUV3-9 proteins level in heads extracts of flies overexpressing either the UAS-*Su(var)3-9-lacI*, the *UAS-hSUV39h1-HA* or *UAS-GFP* construct under the control of the *elav-GAL4* driver. Note that the anti-Su(var)3-9 antibody recognizes both endogenous and LacI tagged protein, but not the human SUV3-9. Anti-HA antibody has been used for specific detection of hSUV39H1-HA. Loading control, Vibrator, Vib. (**B**) Western blots showing the expression levels of TBPH in protein extracts obtained from larval brains (right panel) or adult heads at 3 or 20 dpe (left panel) in absence or in presence of chaetocin 100nM. Note that cheatocin treatment induces a reduction of both the heterochromatic markers H3K9me2 and me3 and a significant increase of TBPH levels. Numbers below each blot represent band quantification, normalized on internal loading (actin or Vibrator, Vib). Similar results were obtained in at least 3 biological repetitions. (**C**) Western Blot showing the TBPH protein levels in *G9a* homozygous mutants ($G9a^{RG5}/G9a^{RG5}$) or eggless mutants (egg^{1473}/egg^{1473}) compared to wild type controls (w^{1118}) in young (3 dpe) fly head extracts. *eggless* mutant flies (egg^{1473}/egg^{1473}) die at 3 dpe and are really sick. Numbers below represent band quantification normalized on internal loading (Vibrator, Vib; average of 4 experiments). (**D**) Western Blot showing the TBPH protein levels in *G9a* homozygous mutants ($G9a^{RG5}/G9a^{RG5}$) compared to wild type controls (w^{1118}) in young (3dpe) or old (20dpe) fly head extracts. Numbers below represent band quantification normalized on internal loading (Vibrator, Vib; average of 4 experiments). (**E**) Climbing assay performed in *G9a* homozygous mutant ($G9a^{RG5}/ G9a^{RG5}$) and in control flies (w^{1118}), at 3 or 20 dpe. Box plot representation of the percentage of flies able to reach the top of a 50 ml tube in 10 seconds after being tapped to the bottom. The line inside the box indicates the median for each genotype and box boundaries represent the first and third quartiles; whiskers are min and max in the 1.5 interquartile range. n > 30 animals for each genotype, in at least three technical replicates. ****p<0.0001, ns= not significant, calculated by one-way ANOVA. Error bars represent SEM. (**F**) qRT-PCR showing *dFus/cabeza* mRNA levels in young (3 dpe; full circles) or old (20 dpe; empty-dotted circles) fly head extracts. Error bars represent SEM of three independent experiments (n = 3; 3 biological replicates and 3 technical replicates). ns: not significant; Mann-Whitney t-test. (**G**) qRT-PCR showing *Su(var)3-9* mRNA levels in young (3 dpe; full circles) or old (20 dpe; empty-dotted circles) fly head extracts. Error bars represent SEM of three independent experiments (n = 3; 3 biological replicates and 6 technical replicates). ** p<0.01; Mann-Whitney t-test. (**H**) qRT-PCR showing *Kdm3*, *Kdm4A* and *Kdm4B* mRNA levels in 3 dpe (full circles) or 20 dpe (empty dotted circles) RNA head extracts from wild type flies (w^{1118}). Error bars represent SEM of three technical replicates (pull of 50 heads). ns=not significant; *** p<0.001; ****p<0.0001 with one-way ANOVA.

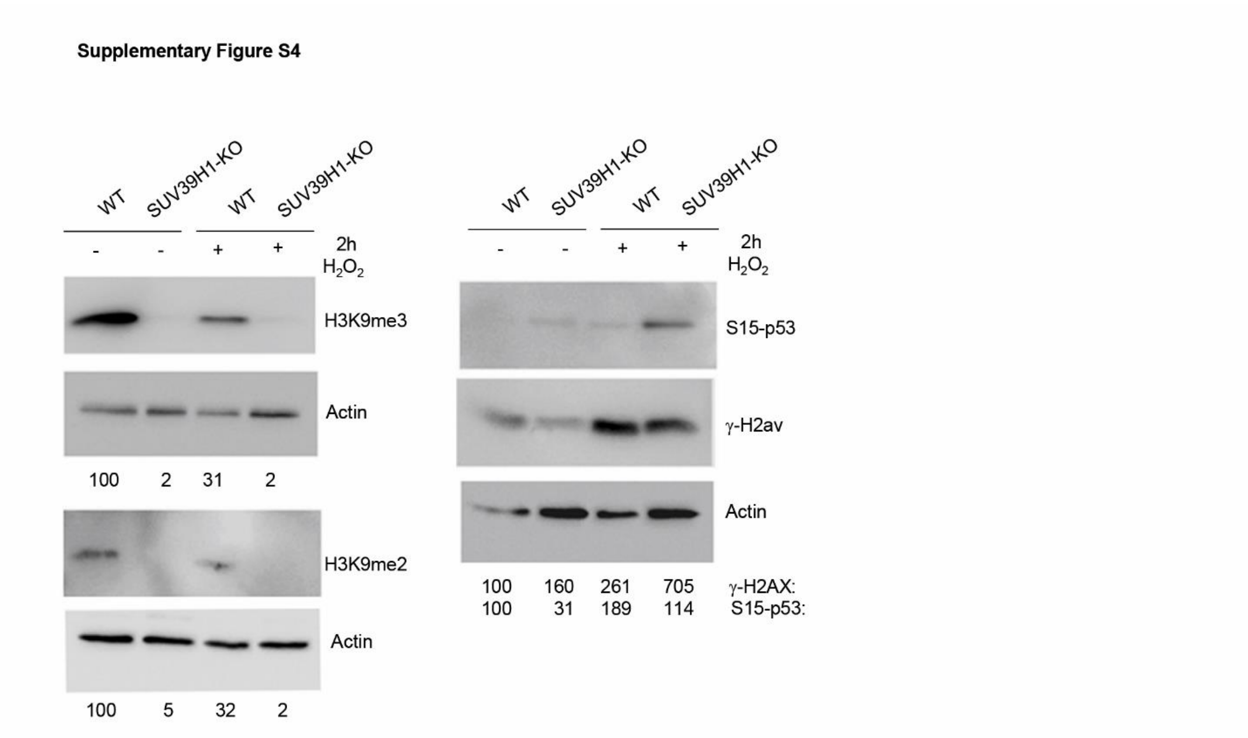

Supplementary Figure S4. HaCaT human cells exhibit age-related molecular signatures upon H_2O_2 treatment

Western blots showing the expression levels of different aging markers (on the right of each blot) in wild type (WT) or *SUV39H1 KO* HaCaT Keratinocytes after (+) or not (-) treatment with H_2O_2 (200mM) for 2 hours (2h). Note that H_2O_2 treatment induces a reduction of both the heterochromatic markers H3K9me2 and me3 and an increase of p53-S15 phosphorylated and γ-H2A.X (positive controls of the treatment). Numbers below each blot represent band quantification, normalized on internal loading (Actin). Similar results were obtained in at least 3 biological repetitions.

Supplementary video V1

Representative video showing a climbing assay performed on w^{1118} at 3 dpe (first tube from left), w^{1118} 20 dpe (middle tube) and *Su(var)3-9* $^{6/1}$ 20 dpe (last tube) flies. The percentage of flies able to reach the top of the 50 ml tube in 10 seconds after being tapped to the bottom is scored to quantify locomotion activity. An average of 15 flies is present in each tube.

Full Blots

Full blot

Figure 1 b

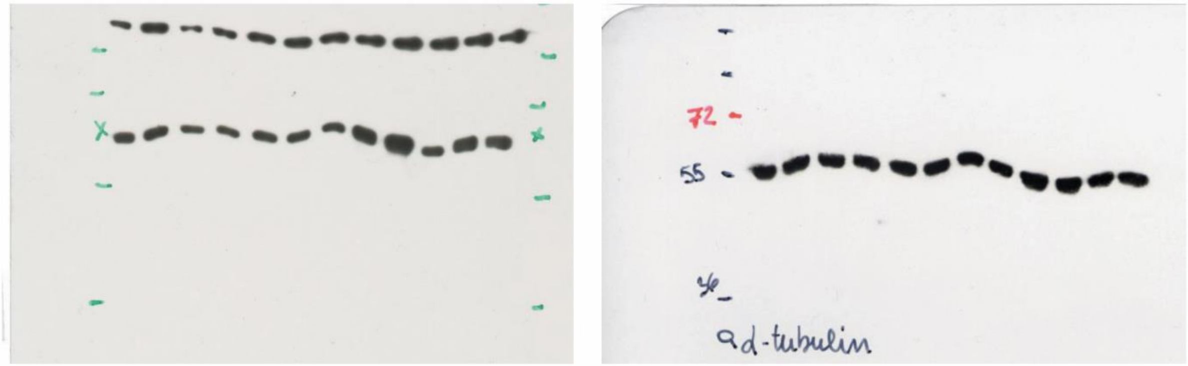

Supplementary Figure S1 b

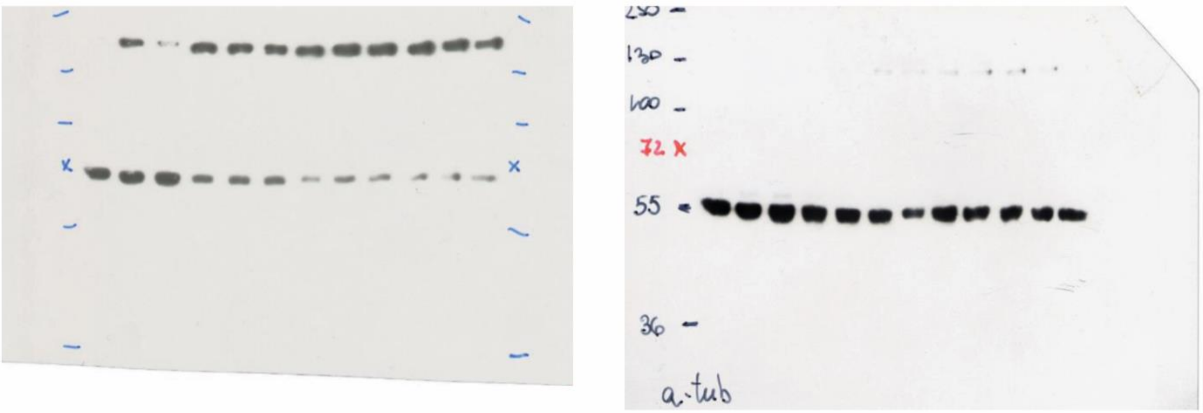

Supplementary Figure S2

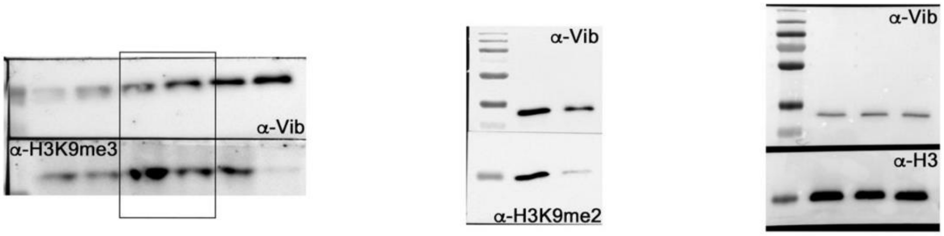

Figure 3

C

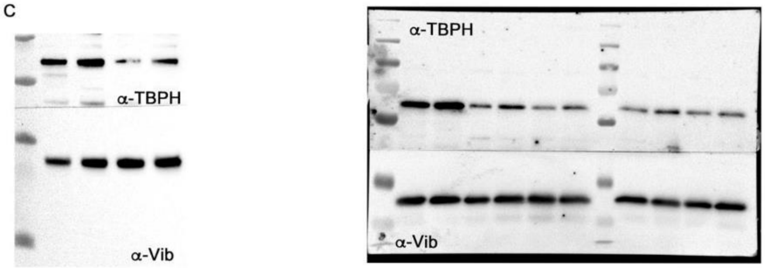

F

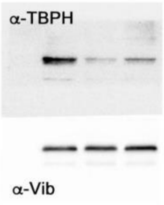

Supplementary Figure 3

A

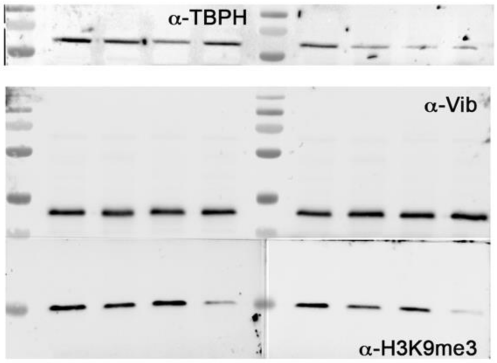

B

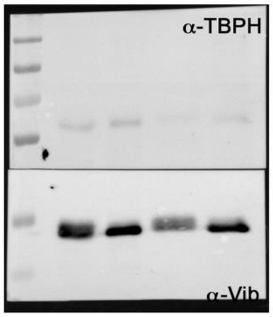

C

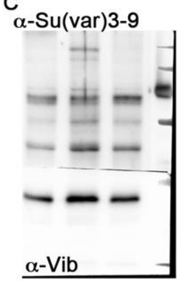

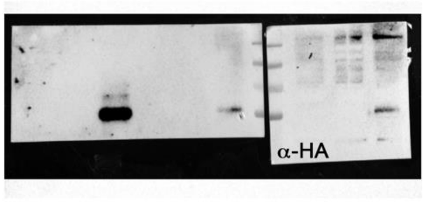

G

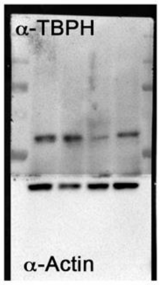

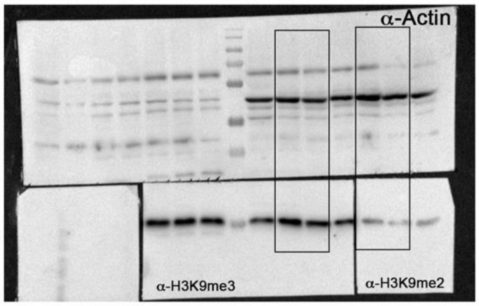

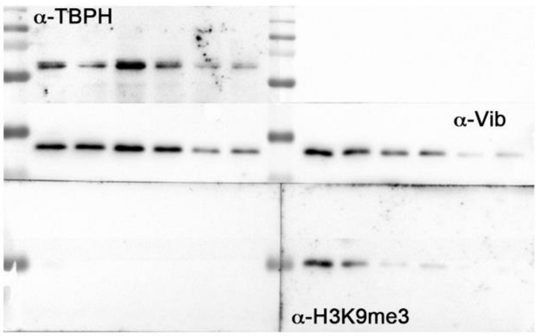

Figure 4A

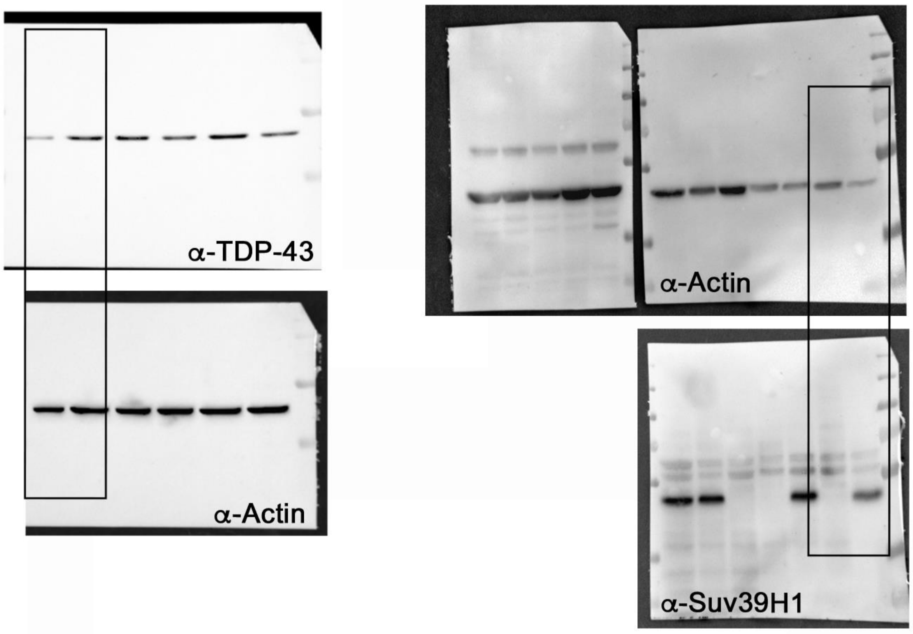

Supplementary Figure 4

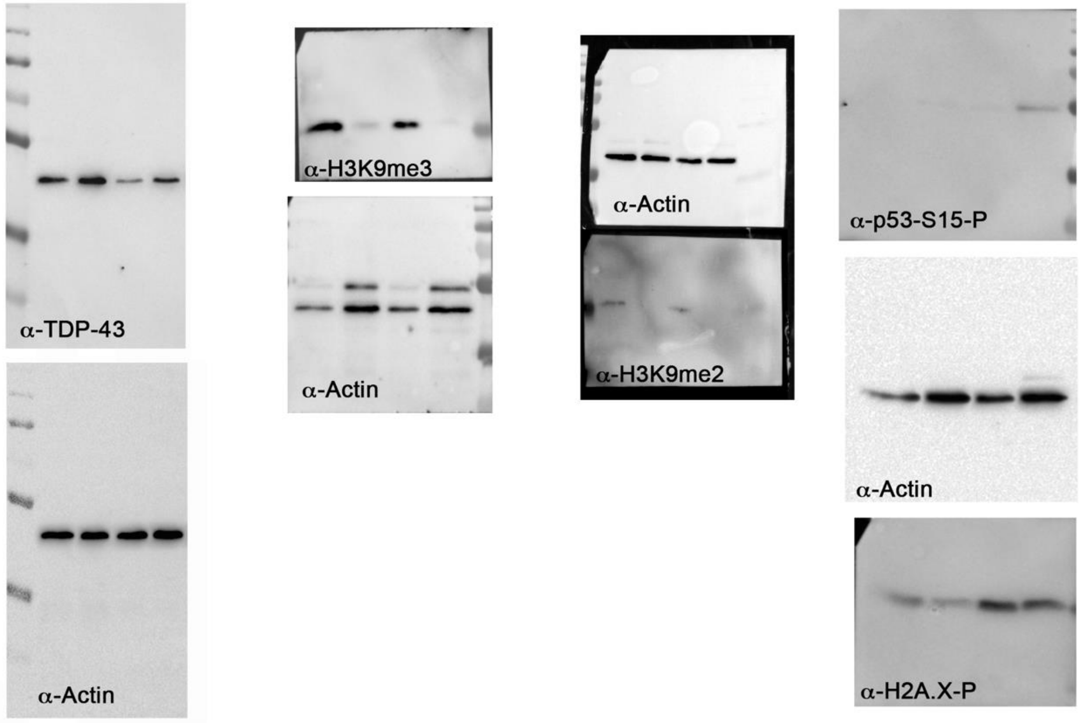

References

1. Cragnaz L, Klima R, De Conti L, Romano G, Feiguin F, Buratti E, et al. An age-related reduction of brain TBPH/TDP-43 levels precedes the onset of locomotion defects in a Drosophila ALS model. Neuroscience. 2015 Dec 17;311:415–21.

2. Coni S, Falconio FA, Marzullo M, Munafò M, Zuliani B, Mosti F, et al. Translational control of polyamine metabolism by CNBP is required for Drosophila locomotor function. Ramaswami M, VijayRaghavan K, Ramaswami M, editors. eLife. 2021 Sep 14;10:e69269.

3. Giaimo BD, Ferrante F, Borggrefe T. Chromatin Immunoprecipitation (ChIP) in Mouse T-cell Lines. J Vis Exp JoVE [Internet]. 2017 Jun 17;(124). Available from: https://www.ncbi.nlm.nih.gov/pmc/articles/PMC5608442/

4. Bosso G, Cipressa F, Moroni ML, Pennisi R, Albanesi J, Brandi V, et al. NBS1 interacts with HP1 to ensure genome integrity. Cell Death Dis. 2019 Dec 13;10(12):1–15.
